# A Psychological Support Intervention to Help Injured Athletes “Get Back in the Game”: Design and Development Study

**DOI:** 10.2196/28851

**Published:** 2022-08-09

**Authors:** Clare L Ardern, Nicholas Hooper, Paul O'Halloran, Kate E Webster, Joanna Kvist

**Affiliations:** 1 Unit of Physiotherapy Department of Health, Medicine and Caring Science Linköping University Linköping Sweden; 2 Sport and Exercise Medicine Research Centre La Trobe University Melbourne Australia; 3 Department of Family Practice University of British Columbia Vancouver, BC Canada; 4 Musculoskeletal & Sports Injury Epidemiology Centre Department of Health Promotion Science Sophiahemmet University Stockholm Sweden; 5 School of Medicine Virginia Commonwealth University Richmond, VA United States; 6 School of Psychology and Public Health La Trobe University Melbourne Australia; 7 School of Allied Health, Human Services and Sport La Trobe University Melbourne Australia; 8 Stockholm Sports Trauma Research Center Department of Molecular Medicine & Surgery Karolinska Institute Stockholm Sweden

**Keywords:** sports, medicine, rehabilitation, sports injury, psychological support, mental health, postoperative medicine, feasibility, eHealth, mobile phone

## Abstract

**Background:**

After a serious knee injury, up to half of athletes do not return to competitive sport, despite recovering sufficient physical function. Athletes often desire psychological support for the return to sport, but rehabilitation clinicians feel ill-equipped to deliver adequate support.

**Objective:**

We aimed to design and develop an internet-delivered psychological support program for athletes recovering from knee ligament surgery.

**Methods:**

Our work for developing and designing the Back in the Game intervention was guided by a blend of theory-, evidence-, and target population–based strategies for developing complex interventions. We systematically searched for qualitative evidence related to athletes’ experiences with, perspectives on, and needs for recovery and return to sport after anterior cruciate ligament (ACL) injury. Two reviewers coded and synthesized the results via thematic meta-synthesis. We systematically searched for randomized controlled trials reporting on psychological support interventions for improving ACL rehabilitation outcomes in athletes. One reviewer extracted the data, including effect estimates; a second reviewer checked the data for accuracy. The results were synthesized descriptively. We conducted feasibility testing in two phases—(1) technical assessment and (2) feasibility and usability testing. For phase 1, we recruited clinicians and people with lived experience of ACL injury. For phase 2, we recruited patients aged between 15 and 30 years who were within 8 weeks of ACL reconstruction surgery. Participants completed a 10-week version of the intervention and semistructured interviews for evaluating acceptability, demand, practicality, and integration. This project was approved by the Swedish Ethical Review Authority (approval number: 2018/45-31).

**Results:**

The following three analytic themes emerged from the meta-synthesis (studies: n=16; participants: n=164): (1) tools or strategies for supporting rehabilitation progress, (2) barriers and facilitators for the physical readiness to return to sport, and (3) barriers and facilitators for the psychological readiness to return to sport. Coping strategies, relaxation, and goal setting may have a positive effect on rehabilitation outcomes after ACL reconstruction (randomized controlled trials: n=7; participants: n=430). There were no trials of psychological support interventions for improving the return to sport. Eleven people completed phase 1 of feasibility testing (technical assessment) and identified 4 types of software errors, which we fixed. Six participants completed the feasibility and usability testing phase. Their feedback suggested that the intervention was easy to access and addressed the needs of athletes who want to return to sport after ACL reconstruction. We refined the intervention to include more multimedia content and support access to and the use of the intervention features.

**Conclusions:**

The Back in the Game intervention is a 24-week, internet-delivered, self-guided program that comprises 7 modules that complement usual rehabilitation, changes focus as rehabilitation progresses, is easy to access and use, and includes different psychological support strategies.

## Introduction

### Background

After a serious knee injury, up to half of athletes do not return to competitive sport [1]. Athletes typically recover sufficient knee function for withstanding the physical demands of playing their sport, but an athlete’s mental state is often the main hurdle to returning to sport [2]; the physical and psychological readiness to return to sport often do not coincide. The fear of reinjury is the most frequent reason reported by athletes who do not return to their preinjury sport or give up their sport after anterior cruciate ligament (ACL) injury or reconstruction [2,3]. Athletes of all ages at all participation levels can have problems with returning to sport after ACL reconstruction [1,4].

The objective of our work was to design and develop an internet-delivered psychological support program for nonprofessional athletes who are recovering from knee ligament (ACL) injury. The aim of the Back in the Game intervention is to deliver on-demand psychological support in parallel with usual postoperative rehabilitation. The goal is to improve athletes’ confidence to return to sport and subsequently improve the return to preinjury sport rate after ACL reconstruction.

### Background Theory

This paper describes the processes for developing an internet-delivered program that provides psychological support for the return to sport to athletes recovering from knee ligament surgery (ACL reconstruction). *Development* refers to the entire process for arriving at an intervention to test in a randomized controlled trial. *Design* refers to the specific decisions we made about the intervention content, format, and delivery mode [5].

**Figure 1:**
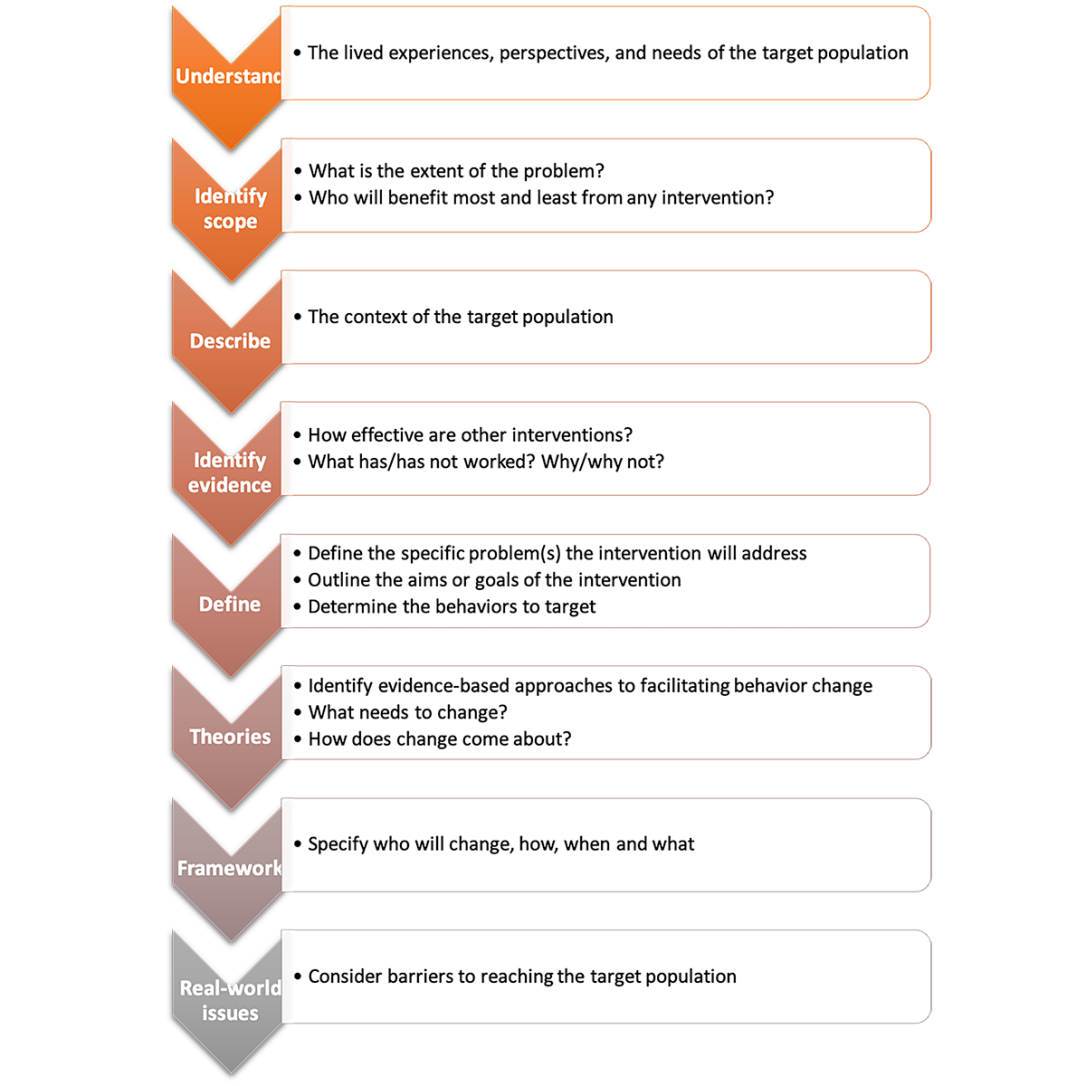
The eight building blocks that underpin our work for developing the Back in the Game intervention.

We adopted a blended approach [5,6] to developing and designing the Back in the Game intervention, which leaned heavily on theory- and evidence-based (United Kingdom Medical Research Council framework for developing and evaluating complex interventions [7]) approaches and target population–based (person-based [8]) approaches. O’Cathain et al’s [5] taxonomy and synthesis guided the development of the eight building blocks that underpin our work (Figure 1).

### Who Benefits From a Psychological Support Intervention?

Athletes with ACL injury expect and desire to return to sport [9-11]. Up to 9 in every 10 recreational athletes expect to return to their preinjury sport after ACL reconstruction [11,12]. Yet, fewer athletes than expected achieve their return to sport goals [1]. Psychological factors have large effects on return to sport outcomes after ACL reconstruction and larger effects on outcomes other than physical function [13,14]. Many psychological factors, including confidence, anxiety, and risk appraisal, are potentially modifiable [1,13]. Greater psychological readiness (a construct that incorporates confidence, emotions, and risk appraisal) is associated with a greater likelihood of returning to the preinjury sport [1]. Therefore, an effective intervention that provides psychological support for the return to sport is likely to be relevant for a broad cross section of athletes.

### Context of the Target Population

A biopsychosocial approach to health and rehabilitation is the dominant paradigm within which health care is delivered in the 21st century [15]. Clinicians recognize that different patients require different emphases on biological, psychological, and social elements and different emphases at different times during a course of treatment. Recovering physical function is vital for achieving return to sport goals; athletes require sufficient physical capacity for coping with the demands of playing their sport, executing their skills as desired, and staying injury-free [16].

The transition from rehabilitation to the resumption of sport after injury can be difficult. Young athletes undergoing rehabilitation get bored and lose motivation during the long rehabilitation period [17,18]. They feel frustrated when rehabilitation does not focus on sports performance [19,20] and are concerned about their body’s ability to cope with the demands of their sport [21]. Anxiety about the consequences of sustaining a knee injury again often besets athletes as they work toward returning to sport [22]. Adding to the challenge, athletes are often discharged from rehabilitation several months before attempting to return to sport. Critically, most athletes lack the support of a rehabilitation clinician during the transition back to sport.

High-quality rehabilitation aims to help athletes gradually regain knee function and physically and mentally prepare for returning to sport. The return to sport occurs along a continuum [23], beginning at injury diagnosis and concluding when the athlete is performing as desired in their chosen sport. Therefore, return to sport support should also be delivered along the same continuum.

## Methods

### Study Design

Our work for developing and designing the Back in the Game intervention involved systematically searching for and synthesizing available literature, designing and developing the intervention, and conducting feasibility testing. We synthesized information on the lived experiences, perspectives, and needs of active people with ACL reconstruction and the effects of psychological support interventions for improving ACL injury rehabilitation outcomes in athletes. After designing and developing the Back in the Game intervention, we conducted a 2-phase feasibility and usability study.

### Lived Experiences, Perspectives, and Needs of Active People With ACL Reconstruction

To understand the issues to be addressed, based on the lived experiences, perspectives, and needs of active people with ACL reconstruction, we systematically searched electronic databases to identify qualitative research exploring the perceptions and experiences of active people with ACL reconstruction. We used the PerSPecTIF (Perspective, Setting, Phenomenon of Interest/Problem, Environment, Comparison, Time/Timing, Findings) framework [24] to construct the research question for a qualitative evidence synthesis, as follows: *“*From the perspective of an active person with ACL reconstruction, in the setting of completing or having completed rehabilitation, how does the phenomenon of biopsychosocial factors during recovery impact on a person’s experiences and perceptions related to recovery and return to sport?*”* The meta-synthesis methods and results from 16 qualitative studies are outlined in Multimedia Appendix 1 [9,10,17,18,25-36].

### Effects of Psychological Support Interventions

To identify evidence of the effects of other psychological support interventions, we systematically searched for research addressing the following question: “What is the efficacy of psychological support interventions for improving ACL injury rehabilitation outcomes in athletes?” We identified and qualitatively summarized the major characteristics of psychological support for athletes and the consequences of providing psychological support during rehabilitation after ACL reconstruction. The aim was to articulate a credible causal explanation for a psychological support intervention that improves the return to sport after ACL reconstruction. The review methods, as well as the specific results and quality assessments from the seven included randomized controlled trials, are outlined in Multimedia Appendix 2.

### Designing and Developing Back in the Game

By combining the results of qualitative and quantitative syntheses, we framed what a psychological support intervention should do and the target behaviors for achieving potential treatment outcomes. We established guiding principles to address key objectives of intervention design and important features that must be addressed to achieve the intervention objectives [8].

### Ethics Approval

This project was approved by the Swedish Ethical Review Authority (approval number: 2018/45-31).

### Feasibility and Usability Study

After designing the first iteration of the intervention, we completed a 2-phase feasibility testing project. Because Back in the Game was a new intervention, we wanted to ensure that it was appropriate and acceptable for athletes after ACL reconstruction. Detailed methods and results for the feasibility and usability study are in Multimedia Appendix 3.

We used maximum variation sampling to recruit 11 participants for phase 1; these participants included physiotherapists, orthopedic surgeons, sports psychologists, researchers from the musculoskeletal rehabilitation field, and people with lived experience of ACL injury. For phase 2, we used strategic sampling to identify and recruit patients with recent ACL reconstruction who were aged between 15 and 30 years at the time of ACL injury, regularly played pivoting and/or cutting sports (eg, football, basketball, and floorball) prior to their injury, and intended to return to their sport. We invited 18 patients, and 7 consented to participate.

In phase 1 of feasibility testing, we focused on addressing practical issues. We sought feedback regarding technical problems with the platform that was used to deliver and display content (ie, feedback not specifically regarding the intervention content). In phase 2, we invited feedback via multiple rounds of interviews on the intervention content, look and feel of the user interface, flow and acceptability of content, frequency of content delivery, and value of the intervention.

## Results

### Lived Experiences, Perspectives, and Needs of Active People With ACL Reconstruction

A total of 16 descriptive themes emerged from the meta-synthesis, which we mapped to the following three analytic themes: (1) barriers and facilitators for the psychological readiness to return to sport, (2) barriers and facilitators for the physical readiness to return to sport, and (3) tools or strategies for supporting rehabilitation progress.

Athletes at all levels (ranging from amateur to professional) shared common perceptions and lived experiences of rehabilitation after ACL reconstruction (Table S1 in Multimedia Appendix 1 [9,10,17,18,25-36]). Athletes wanted to play sports and saw playing sports as central to their self-identity [25,26]. They felt happy when they were playing sports [9]. However, the experience of ACL injury had often irrevocably changed how they thought of themselves and their capacity to contribute to society [27].

Anxiety and low confidence were dominant emotions. Athletes felt scared, uncertain, frustrated, and hopeless at different times during recovery and when returning to sport. Sometimes, athletes avoided tasks or activities (eg, sport-specific movements) because they lacked confidence in their knee [9,10,17,18,26-34]. Athletes drew support, feedback, encouragement, and reassurance from people they trusted. Previous experiences of sports injury also helped athletes know what was required to recover and return to sport [17,18,25-33,35,36].

Athletes judged that quality rehabilitation programs included strategies that help build their physical and mental capacity for participating safely in sports again [9,10,17,28,29,32,34,36]. Textbox 1 summarizes the key aspects of athletes’ perspectives, lived experiences of rehabilitation, and needs after ACL reconstruction that informed the development and design of the intervention (Table S2 in Multimedia Appendix 1 [9,10,17,18,25-36] provides a detailed summary).

**Evidence summary: athletes’ lived experiences. Themes 1, 2, and 3 were barriers and facilitators for the psychological readiness to return to sport, barriers and facilitators for the physical readiness to return to sport, and tools or strategies for supporting rehabilitation progress, respectively.**

**Athletes’ lived experiences**

- Athletes wanted to play sports and felt happy when they were playing sports (theme 1).
- Athletes felt nervous and worried about sustaining another knee injury (theme 1).
- Athletes lacked self-confidence, self-efficacy, and self-esteem during rehabilitation (theme 1).
- Athletes knew there were risks associated with returning to sport and took active steps to manage the risk of new injury (theme 2).
- Injured athletes wanted the following (theme 3):
  Strategies to help manage pain and injury risk
  An understanding of their injury and what was required for recovery
  Support from important people (eg, family, friends, physiotherapist, and coach)
  Help with setting goals
  Regular feedback

### Effects of Psychological Support Interventions

We identified 7 randomized controlled trials for inclusion in the quantitative synthesis of the effects of psychological interventions in ACL rehabilitation (Figure S1 in Multimedia Appendix 2). The psychological support interventions with the most research attention were imagery, relaxation, and goal setting. Effect estimates are summarized in Table S1 in Multimedia Appendix 2.

Psychological skills training that targets coping strategies, relaxation, and goal setting might work for improving physical impairments and psychological outcomes after ACL reconstruction. It is uncertain whether imagery/visualization is helpful for injured athletes. Textbox 2 outlines treatment approaches with the potential to improve health outcomes and the potential for inclusion in a new psychological support intervention.

**Evidence summary: treatment approaches.**

**Treatment approaches**

- Watching others who have experienced the injury and rehabilitation before completing rehabilitation exercises
- Learning about the ways people coped with the mental and physical challenges of rehabilitation
- Watching a carefully curated set of images designed to induce positive psychological responses toward injury and rehabilitation
- Guided relaxation
- Guided goal setting
- Guided imagery

### Designing and Developing Back in the Game

A psychological support intervention for helping athletes return to sport after injury should deliver practical tools or strategies that athletes can use to complement physical rehabilitation (Textbox 3). The intervention should (1) help injured athletes understand their injury and what is required for recovery; (2) support athletes in setting realistic goals and provide regular feedback on progress toward athletes’ goals; (3) teach athletes strategies for managing fear and anxiety, boosting low confidence and self-efficacy, and maintaining motivation and athlete identity; and (4) support athletes in establishing lifelong habits and staying healthy while playing sports. We established 5 guiding principles [8] for intervention design (Textbox 4).

**Figure 2:**
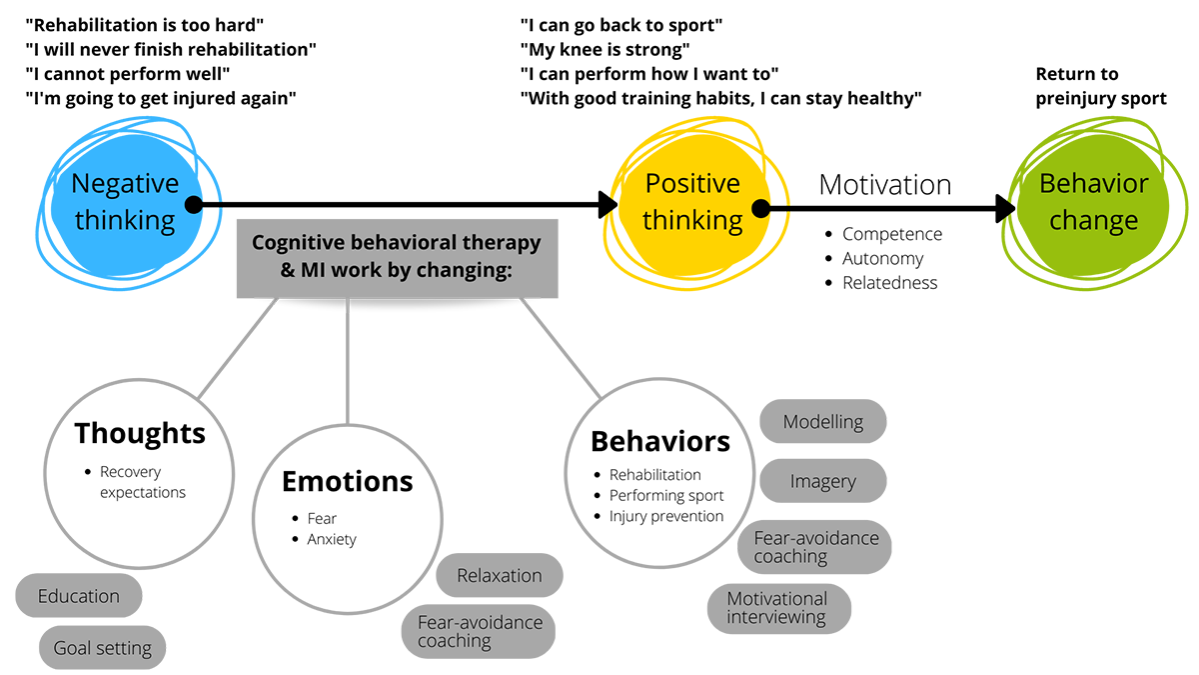
Back in the Game is grounded in cognitive theory and self-determination theory and aims to facilitate return to sport behavior change. Topics that are listed in grey ovals (adjacent to "Thoughts," "Emotions, and "Behaviors") are the key contents of the intervention modules. MI: motivational interviewing.

Back in the Game delivers cognitive behavioral therapy plus motivational interviewing to help athletes identify negative thoughts about the behavior of playing sports and reframe negative thoughts into positive thoughts to promote behavior change (Figure 2). The rationale for the content and delivery mode is that supporting athletes in achieving psychological readiness to return to sport while they complete usual postoperative rehabilitation helps them to more successfully transition back to the preinjury sport.

**Evidence summary: behaviors to target with a psychological support intervention for athletes with anterior cruciate ligament reconstruction.**

**Behaviors to target**

- Fear avoidance
- Setting goals
- Understanding how to recover well from injury
- Safely participating in sport
- Ongoing injury prevention
- Practicing psychological support skills (imagery/visualization, relaxation, etc)

**Evidence summary: guiding principles for the structure and delivery of the Back in the Game intervention.**

**Guiding principles**

- Complement usual rehabilitation care
- Change focus as rehabilitation focus changes
- Self-guided and easy to access
- Unobtrusive and not burdensome to use
- Include a range of psychological techniques/strategies (something to meet everyone’s needs)

**Figure 3:**
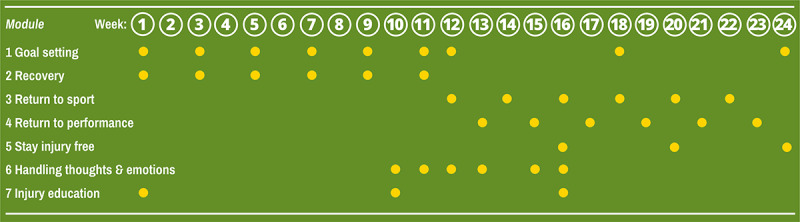
Summary of the seven self-directed modules (covering psychological skills, psychoeducation, and principles of motivational interviewing) of the Back in the Game intervention. Each dot represents how often the user is prompted to complete a task in each module.

Back in the Game is a 24-week intervention that commences in the first week following ACL reconstruction. A total of 7 modules (Figure 3) are delivered in parallel with usual rehabilitation care. The intervention mirrors the progression of rehabilitation, functions as a stand-alone eHealth intervention, and does not require monitoring or input from the clinician responsible for delivering rehabilitation. Tasks are delivered in a progressive fashion (ie, users receive a notification to log in and take action in the app every time there is new content available and up to two reminders to access the content each week) and are tailored to the stage of face-to-face rehabilitation. Athletes choose, from a menu of different tasks, the task that best suits their needs during each intervention session.

**Figure 4:**
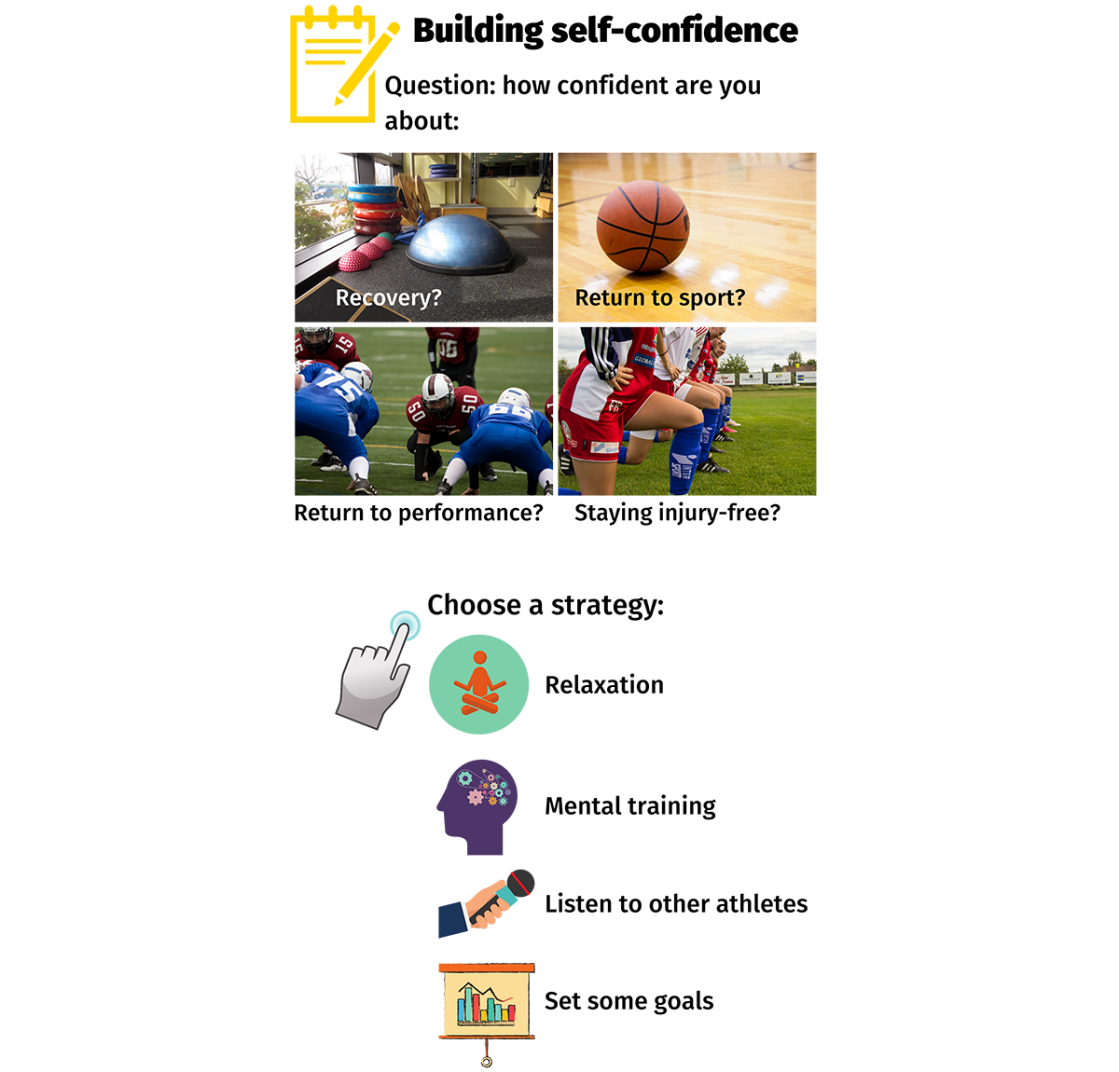
Overview of the "Recovery," "Return to sport," "Return to performance," and "Staying injury-free" modules plus linked cognitive behavioral therapy tasks (strategies). This screenshot is presented in the content overview video that accompanies the app introduction, which users receive when they register.

**Figure 5:**
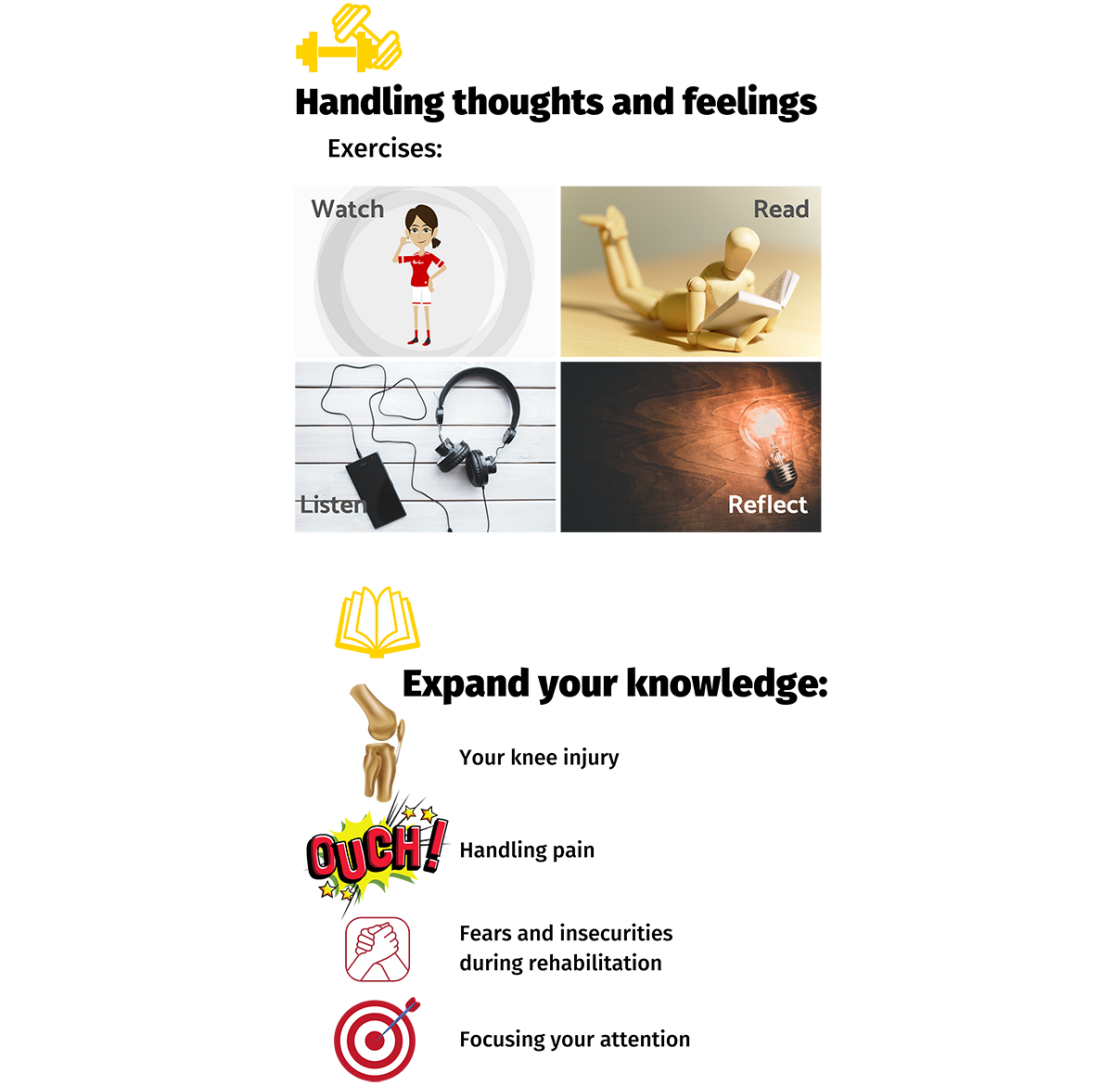
Overview of the "Handling thoughts & emotions" and "Injury education" modules. This screenshot is presented in the content overview video that accompanies the app introduction, which users receive when they register.

The motivational interviewing delivered in Back in the Game focuses on athletes’ confidence for recovering from their knee injury, returning to sport, returning to performance, and staying injury-free (Figure 4). Linked to each motivational interviewing question are strategies for building athletes’ psychological skills in relaxation, mental imagery, education, and goal setting (Figure 4). The modules *Handling thoughts & emotions* and *Injury education* comprise tailored content that athletes can watch, read, and listen to (Figure 5).

### Feasibility and Usability Study

We worked with the content delivery platform vendor (Briteback AB, Norrköping, Sweden) to complete additional engineering tasks and implement bug fixes in response to the feedback we received in phase 1. Table S1 in Multimedia Appendix 3 details the specific bugs and bug fixes. In phase 2, we identified aspects of the intervention that were satisfactory and did not require changes (Table S4 in Multimedia Appendix 3) and aspects that required specific changes/actions (Table S5 in Multimedia Appendix 3).

Users (1) thought that the intervention would add value to their physical rehabilitation, (2) found appealing content with appropriate flow, (3) appreciated receiving notifications and reminders to engage with the intervention, and (4) said that the goal-setting module was helpful. Users (1) wanted more support for starting the intervention, (2) wanted to better understand what they had to do, and when and why they had to do certain tasks, and (3) wanted more feedback on their progress through the intervention. In response to the feasibility testing results, we added additional video and infographic content and strengthened the system for providing tailored progress reports and feedback.

## Discussion

### Principal Findings

Back in the Game employs a mix of psychological skills, psychoeducation, and motivational interviewing. Our blended approach to developing the intervention ensured that the content choices were informed by the prominent emotions that athletes said they felt (confidence and fear), the strategies that were tested in previous research (goal setting, imagery, relaxation, and behavior modeling), and what athletes said they needed (support for setting goals, managing pain and injury risk, understanding their injury and what was required for physical and mental recovery, and receiving feedback on their recovery progress). A person-based approach [8] informed the decisions we made about the intervention content, format, and delivery mode (designing the intervention). This approach allows designers to understand what to do to design an attractive intervention that (1) addresses end users’ needs and (2) is feasible to implement [8].

Internet-delivered psychological support works best when users feel engaged in their mental health support in real time, when the intervention employs a user-friendly interface that prioritizes multimedia, and when the intervention structure encourages users to engage in self-monitoring [37]. We embraced the recommendations for designing mental eHealth interventions and established key design principles for guiding our work.

We recommend that athletes use the Back in the Game intervention for a minimum of 30 minutes every week. It is rare for athletes to return to a pivoting sport before 6 postoperative months, and athletes are actively encouraged to delay returning to unrestricted participation in a pivoting sport for at least 9 months [16]. The intervention is designed to help athletes prepare for the return to sport; we do not expect athletes to return to their preinjury sport during the 24-week intervention period. However, we expect that some athletes will participate in a modified sport (eg, no contact and no direction changes) by the end of the intervention period.

People’s beliefs, perceptions, plans, and interpretations influence their behaviors and emotions and determine how the world influences them [38,39]. Cognitive theory underpins cognitive behavioral therapy. The aim of cognitive behavioral therapy is to transform negative thoughts/thinking into positive thoughts/thinking by changing a person’s thoughts, emotions, and behaviors. Cognitive behavioral therapy targets a person’s interpretations and beliefs. Uncovering and changing negative thinking patterns boosts a person’s self-motivation to engage in a health-promoting behavior [38]. This is the central theory underpinning the Back in the Game content. Based on the combination of cognitive theory and self-determination theory [40], we propose that when one has positive thoughts about a behavior, the motivation to engage in this behavior is enhanced, ultimately boosting the likelihood of engaging in and sustaining the behavior.

We recognized that motivation was highlighted in athletes’ lived experiences of recovering from ACL reconstruction, and we incorporated self-determination theory into our framework for Back in the Game. The motivation to change one’s behavior is strongest when one feels that the behavior is self-determined [40]. For the return to sport, an athlete’s self-motivation to engage in a sport (ie, fulfilling return to sport goals) is driven by the following three key elements: a sense of personal control over what happens (autonomy), a belief that one has the skills and knowledge to succeed (competence), and support for achieving one’s goals (relatedness) [40].

### Real-world Issues

Back in the Game is a self-directed psychological support intervention that was designed to be available on-demand and be delivered via the internet (smartphone app or website). An intervention that complements physical (face-to-face) rehabilitation might be an effective tool for overcoming potential barriers to delivering effective psychological support to athletes during and after rehabilitation [37,41]. We speculate that geography, cost, and stigma are barriers that might prevent athletes from accessing the psychological support that they need to return to sport after injury. An eHealth intervention can deliver content to athletes, regardless of where they live or train, at low (or no) financial cost to the athletes, and the athletes do not need to disclose to anyone that they are accessing the psychological support content provided by an eHealth app.

For at least 2 decades, clinicians and health researchers have developed and delivered psychological treatments via the internet [42]. Digital interventions can be as effective as in-person visits to a psychologist, can deliver sustained benefits, and are probably cost-effective [43]. Using digital interventions to complement face-to-face sports injury rehabilitation is an area of growing research interest [44-46]. eHealth technology facilitates the low-cost, on-demand delivery of psychological support to injured athletes [41-43]. Smartphones are an accessible platform [47] that can be used to deliver evidence-based strategies for improving the confidence to return to sport that athletes can access anywhere at any time.

### Conclusion

We describe a multifaceted approach to developing a complex eHealth intervention that considers an athlete’s lived experience and context, previous work in the field, a theoretical rationale for the intervention, and input from end users to ensure an appropriate and acceptable final product.

The Back in the Game intervention is a 24-week, internet-delivered program that covers psychological skills, psychoeducation, and principles of motivational interviewing. The self-guided intervention complements usual rehabilitation, changes focus as rehabilitation progresses, is easy to access and use, and includes different psychological support strategies. End users suggested that the Back in the Game intervention met our objective of developing a psychological support intervention that was easy to access, focused on the return to sport, and complemented usual postoperative rehabilitation (ie, the intervention was appropriate and acceptable for the target population). End users had a generally positive attitude toward the intervention.

The results from the feasibility and usability testing phase confirmed that Back in the Game is an intervention that is worth testing in a definitive randomized controlled trial. The Back in the Game trial [48] commenced recruitment in 2019, and it aims to recruit 220 young athletes with ACL injury to test the effects of the Back in the Game intervention on the return to preinjury sport and level after ACL reconstruction.

## Appendix

Multimedia Appendix 1 Lived experiences, perspectives, and needs of active people with anterior cruciate ligament reconstruction.

Multimedia Appendix 2 Effects of psychological support interventions for improving anterior cruciate ligament injury rehabilitation outcomes in athletes.

Multimedia Appendix 3 Feasibility and usability of the Back in the Game intervention.

## Abbreviations

ACL: anterior cruciate ligament
PerSPecTIF: Perspective, Setting, Phenomenon of Interest/Problem, Environment, Comparison, Time/Timing, Findings
