# Supplementary material for: A Psychological Support Intervention to Help Injured Athletes “Get Back in the Game”: Design and Development Study
Source: JMIR Form Res. 2022 Aug 9;6(8):e28851. doi: 10.2196/28851 (PMC9399889; doi:10.2196/28851)
Supplement: Multimedia Appendix 1 [file formative_v6i8e28851_app1.pdf]

APPENDIX 1. Understanding Injured Athletes' Lived Experience, Perspectives and Needs

In this section, we describe the methods and results addressing the question: *From the perspective of an active person with ACL reconstruction, in the setting of completing or having completed rehabilitation, how does the phenomenon of biopsychosocial factors during recovery impact on a person's experiences and perceptions related to recovery and return to sport?*

Search Approach

We searched the MEDLINE, EMBASE AND SPORTDiscus electronic databases from inception (updated on 22 April 2020) using the key words "anterior cruciate ligament" AND ["interview" OR "qualitative"]. We deliberately used broad search terms because searching for qualitative syntheses is notoriously challenging.[1, 2] We supplemented the search with forward citation tracking (using Google Scholar), related paper searching, and hand searching the reference lists of included records.

Study Selection

We selected studies that examined the opinions of people with ACL reconstruction about at least 1 issue related to returning to sport after ACL reconstruction, and that were reported in English language. We excluded studies that used mixed-methods, semi-quantitative or quantitative methods.

One researcher conducted the database search; a second researcher selected the articles. Records were exported to EndNote X9, and duplicates were removed before screening. Article selection occurred in two steps. First, the researcher screened the titles and abstracts of all records. Where it was unclear from the title and abstract whether an article should be included, the article was obtained in full text and screened. Once the final list of included articles was established, a second researcher checked the list and agreed on the final list.

Data Extraction

One researcher extracted study and participant characteristics (sample size, sex, age, country, and population), and key results statements from each included study; a second reviewer cross-checked the accuracy of the data extraction. The key results statements were organised in a table ready for coding to commence.

Data Synthesis

We used a thematic synthesis approach [3, 4] to gather, code and summarise information, and identify key themes and a novel interpretation of the body of qualitative literature.[3, 4] Coding was an iterative process through which we established and refined a thematic framework for synthesising information. The data extraction, coding and synthesis approach occurred in parallel, concurrently refining and developing the synthesis framework as themes emerged. Quotes from study participants and the descriptions of key study results informed new themes from the available data.

There were 4 steps in the synthesis process:

1. A researcher read each article and completed free line-by-line coding of the results, which was cross-checked by a second researcher.
2. Two researchers collaborated to establish new descriptive themes from the line-by-line coding.
3. One researcher wrote a summary of the synthesised results, organised by the 16 descriptive themes; a second researcher cross-checked the summary.
4. Two researchers distilled the descriptive themes to new analytic themes that addressed the aim of reviewing the literature.

We consistently reviewed and cross-checked the descriptive theme labels so that earlier coding could guide later coding, and we could update earlier coding as new information emerged. Finally, we reviewed all descriptive themes and wrote descriptive summaries based on the line-by-line coding. Two reviewers worked on the coding the synthesis: line-by-line coding and descriptive themes were identified by each reviewer independently; one

reviewer wrote the descriptive summaries, and the second reviewer cross-checked the descriptive summaries for accuracy. We present the final results in summary tables.

Results

We identified 240 records. After deleting duplicate records and screening, there were 16 qualitative studies included (Figure S1) that reported the experiences and opinions of more than 164 participants (1 study did not report how many participants with ACL reconstruction were interviewed). The participants were mainly professional or non-professional athletes, predominately in their teenage or young adult years. There were 77 female participants and 85 male participants (2 studies did not report sex). Studies were conducted in high income countries in Australasia (*n* = 3), Europe (*n* = 8) and North America (*n* = 5) (Table S1).

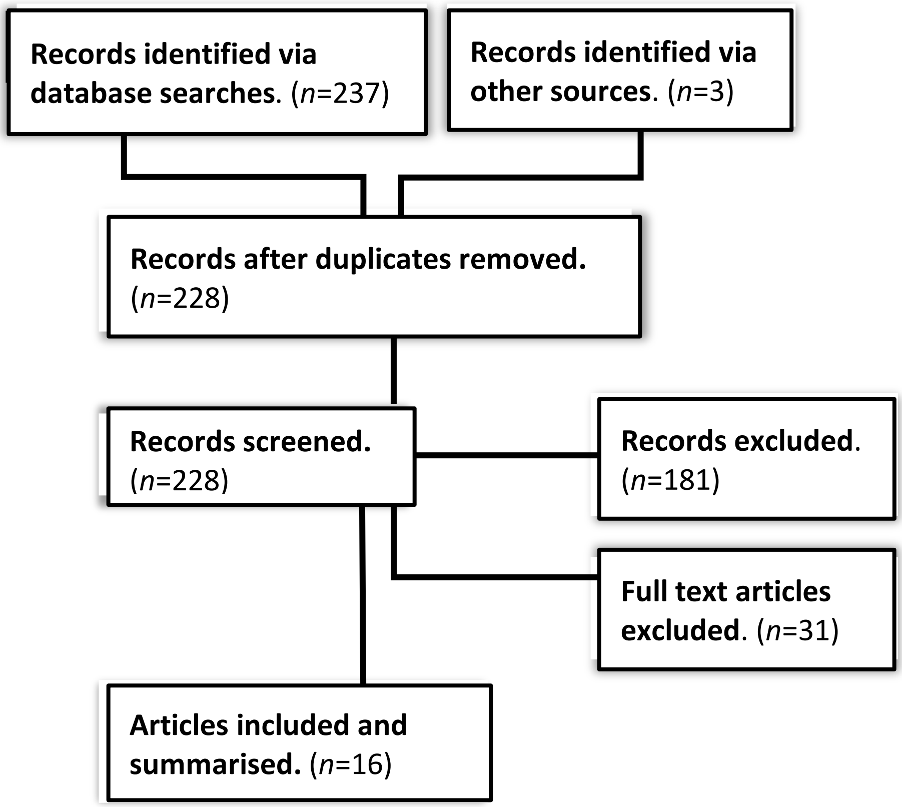

Figure S1. Identifying and selecting qualitative studies exploring the perceptions and experience of people with ACL reconstruction during rehabilitation and return to sport.

Table S1. Summary of qualitative study characteristics

Study identifier	Country	Population	n	Sex (F:M)	Age
Thing 2006[5]	Denmark	Non-professional handball players completing rehabilitation after ACL reconstruction	17	17:0	19-33 years
Heijne 2008[6]	Sweden	Non-professional pivoting sport athletes, 12-21 months after ACL reconstruction	10	1:9	23-41 years
Carson 2012[7]	England	Professional rugby union players during rehabilitation and transition back to sport	5	0:5	18-27 years
Tjong 2013[8]	Canada	Recreational (*n* = 20), high school (*n* = 4), university (*n* = 6) and professional (*n* = 1) athletes at least 2 years after ACL reconstruction	31	9:22	18-40 years
Nordahl 2014[9]	Sweden	Elite alpine skiers from a selective sports high school, 5 years after ACL reconstruction	5	2:3	16-19 years
Gill 2016[10]	England	University students	NR	NR	NR
Johnson 2016[11]	Sweden	Elite football players, 4-9 months after a first-time ACL reconstruction	8	13:0	25-35 years
Carson 2017[12]	England	Professional rugby union players during rehabilitation and transition back to sport	5	0:5	18-27 years
Burland 2018[13]	United States	Athletes who were active in competitive sport before ACL injury, at least 1 year after ACL reconstruction	12	6:6	16-44 years
Scott 2018[14]	New Zealand	Recreational pivoting sport athletes, 8-36 months after ACL reconstruction	9	4:5	21-37 years
DiSanti 2018[15]	United States	High school pivoting sport athletes with ACL reconstruction who had not returned to sport	10	7:3	15-18 years
Conti 2019[16]	Italy	Professional basketball players who had returned to basketball after ACL reconstruction	10	0:10	22-36 years
Paterno 2019[17]	United States	Youth pivoting sport athletes who had and had not returned to sport	10	4:6	11-19 years
Sole 2019[18]	New Zealand	Competitive pivoting sport athletes up to 5 years after ACL reconstruction	9	5:4	18-26 years
Kunnen 2020[19]	Australia	Amateur (*n* = 17), national (*n* = 2) and international (*n* = 2) football players who had returned to sport	21	9:12	19-51 years
Truong 2020[20]	Canada	Youth athletes up to 2 years after ACL injury, who had returned to sport	7	NR	15-19 years

Note. NR, not reported; [7] and [12] reported on the same sample.

Meta-Synthesis

Three analytic themes emerged from the studies of athletes' experiences, perceptions and needs, based on 16 descriptive themes (Table S2):

1. Barriers and facilitators for *psychological readiness to return to sport*
2. Barriers and facilitators for *physical readiness to return to sport*
3. *Tools or strategies* to support rehabilitation progress

A psychological support intervention to help athletes return to sport after injury should include content targeting barriers and boosting facilitators for physical and mental readiness to return to sport, and aim to provide practical tools or strategies that athletes could use in parallel with their physical rehabilitation programme.

Table S2. Athletes' perceptions and experiences related to recovery and return to sport after ACL reconstruction

Descriptive theme (review finding)	Studies contributing to the review finding	Exemplar quotation 1	Exemplar quotation 2	Analytic theme
**Education**				
Early in the rehabilitation period, athletes wanted to learn about what was required for successful outcomes after injury. They wanted to learn coping strategies that could help their recovery, including how to cope with pain and how to manage risk. Athletes saw the clinician as a guide to what they (the athlete) needed to do to reach their return to sport goals.	[5, 6, 10, 12, 16, 17, 20]	A parent speaking about her son's physical therapist: "[the physical therapist] was really instrumental in helping him [her son] understand his injury," prior to working with the physical therapist her son "was really, really confused".[17]	"I'm learning more and more about the injury and the best methods to rehabilitate. The medical team probably hate it because I'm asking so many questions."[11]	Tools or strategies
**Communication**				
Constructive communication with others, including coaches and clinicians, helped athletes stay motivated and confident about their recovery and return to sport. Feeling uncertain hindered athletes' progress, and clinicians could help by clearly articulating the pathway and timeline for recovery.	[7, 11, 14, 15]	"Our coach has been really good; he has listened to me and often asked how I felt and so on."[11]	"The coach, [physiotherapist] and I all sat down to look at the schedule and decide when it was best for me to return to the line up. They wanted to ease me back in gently and we looked at how long I would play for."[7]	Tools or strategies
**Information**				
Athletes wanted clear information about how long it would take to recover and return to sport. They also wanted information to help understand ACL injury and what	[11, 14-17, 20]	"why it's happened, what's happened, and what's going to happen";	"I remember a few weeks before my first competition, my	Tools or strategies

Descriptive theme (review finding)	Studies contributing to the review finding	Exemplar quotation 1	Exemplar quotation 2	Analytic theme
rehabilitation would entail. When athletes understood their situation and what was required to recover, they felt more confident about the prognosis for recovery and return to sport. Having access to appropriate resources at the appropriate time during rehabilitation (eg gymnasium, clinicians, exercise programmes) was important, and athletes knew they needed to take proactive steps to ensure they stayed healthy after returning to sport.		"she just had a wealth of knowledge…I knew why I was doing something, or I knew that this happened with every ACL patient, and I didn't have to worry about it."[14]	athletic coach had spent some time giving me information on the possible physical conditions of an athlete who comes from a long time injury and comes back to compete; it was very useful for me to create ideas and expectations about what I would have found on the court."[16]	
**Support from others/social support**				
Athletes drew on social support from different places, including family, friends, rehabilitation clinicians, coaches, and teammates/peers. Athletes viewed social support as a critical part of their rehabilitation and vital for maintaining motivation and confidence to return to sport. Athletes drew support, feedback, encouragement and reassurance from people they trusted.	[5, 7, 9-18, 20]	"Having around people who support you like family and friends, is really comforting and helps you to feel not alone."[16]	"I think it would help a lot to have some sort of support system…with somebody else [who] has done it and has recovered in…similar conditions as you…so like for me, it would be nice the first time I tore my ACL to have, say, a basketball player [who is] now playing college or something like that who has torn his ACL, [been through] the	Tools or strategies

Descriptive theme (review finding)	Studies contributing to the review finding	Exemplar quotation 1	Exemplar quotation 2	Analytic theme
			recovery process, and is back to playing. Just for me to see that it works."[15]	
**Feedback**				
Regularly measuring function and seeing it improve helped athletes stay motivated during rehabilitation. Athletes trusted rehabilitation clinicians to provide appropriate and timely feedback on rehabilitation progress. Athletes described receiving feedback as a key pillar of quality rehabilitation after ACL injury.	[6, 12, 16, 17, 19]	"I have seen a gradual improvement in my range of movement, so I am positive I am improving all the time."[12]	"I wish I would have a chart of where I should be…so I could know what I'm shooting for…"[17]	Tools or strategies
**Setting and achieving goals**				
Setting goals helped athletes stay motivated and adequately prepare physically and mentally to return to sport. Some athletes had good support from clinicians to set rehabilitation and return to sport goals that mattered to them, while others received insufficient support. Athletes wanted support from clinicians to set goals, and saw goal setting and a structured approach to charting progress as a hallmark of quality rehabilitation.	[6, 7, 10-12, 15-17, 19]	"My outlook and my goal is to be back in 6 months after the operation…we play Champions League then. I will be physically and mentally ready at that time."[11]	"[Coach] said I needed to come in field and get involved on five separate occasions. It was good to have this target, as I could have easily stayed out on the wing and not been involved."[12]	Tools or strategies
**Coping strategies**				
Coping with injury was facilitated by positive attitudes and personality traits (eg optimism, patience, determination) and cognitive processes like problem-solving, goal setting and rational thinking. Coping with injury was central to effectively completing rehabilitation.	[9, 16, 18]	"In my opinion it is important to and useful to have a life outside of basketball. Having other interests, hobbies, and friends helped me to not be focused on the injury,	"If you are the kind of person with an optimistic outlook, as I see myself, it helps you experience greater focus and less concern	Tools or strategies

Descriptive theme (review finding)	Studies contributing to the review finding	Exemplar quotation 1	Exemplar quotation 2	Analytic theme
		especially when I was feeling down."[16]	about the rehabiilitation process."[16]	
**Staying injury free**				
Fear about sustaining another ACL injury was one of the most prominent emotions for athletes during rehabilitation and when returning to sport. Accepting that there was risk inherent in playing sport, and learning how to manage that risk, including learning strategies to help prevent new ACL injuries, were important to athletes. Athletes recognised that they were responsible for continuing strategies like injury prevention exercises after returning to sport. Athletes said that good rehabilitation programmes included strategies to help build their physical and mental capacity to participate safely in sport.	[5, 8, 10, 14, 15, 18-20]	"My knee was pain free. I could run as far and fast as I could pre-op. I was able to train fully with contact and cut and turn movements for 3 months before I felt confident and comfortable being able to play. Most importantly, I didn't have any knee awareness, and I wasn't scared that the movement that caused my injury would cause me to reinjure it."[19]	"I'm terrified of injuring myself again; I also just think how fluky my step was…I planted my foot, and I tore my ACL. So, to think about that…is a little bit nerve-wracking."[15]	Barriers and facilitators for physical readiness to return to sport
**Capacity of one's body to perform in sport**				
Athletes were nervous about whether they could perform well in their sport after returning from injury, and felt personal responsibility for their progress towards full recovery. Athletes felt confident about their rehabilitation preparing them for the demands of performing their sport, but they were concerned about sustaining another injury while playing sport. Performing sport-specific movements and tasks during rehabilitation, and using visualisation/mental imagery, helped athletes prepare	[6-9, 13, 15, 16, 19]	"I'm just worried that I am not going to be as competent as I was and that's going to reflect in my playing, because I feel if you're really timid in your playing, then you're going to get hurt again."[15]	"I'm still nervous about how my fitness will hold up"; "I am very excited about the prospect of playing a game. I'm nervous about how I will perform rather than how my knee will be."[12]	Barriers and facilitators for physical and psychological readiness to return to sport

Descriptive theme (review finding)	Studies contributing to the review finding	Exemplar quotation 1	Exemplar quotation 2	Analytic theme
psychologically to perform well when they returned to their sport.				
**Emotions**				
Athletes felt scared, uncertain, frustrated, and hopeless at different times during recovery. Sometimes they avoided activities because of fear or lack of confidence. Athletes' fear and anxiety was typically about getting the injury again, having to go through rehabilitation again, the long-term consequences of injury and whether they could perform well again. Athletes felt happy and free when playing their sport.	[5-11, 13-15, 17, 19, 20]	"Well I was happy to stand on skis again, and in the beginning it was fun to go out in the snow for the first time after my injury"[9]	"If you got into a bad car accident on a highway, then you may not drive on that road again. That's how I feel about my ACL."[8]	Barriers and facilitators for psychological readiness to return to sport
**Expectations**				
Athletes expected to learn realistic timelines for recovery and return to sport from the clinician. Feeling uncertain about their progress hindered mental preparation for returning to sport, but when athletes understood the process, they felt confident and prepared. Athletes' expectations affected how ready they felt to contribute to decisions during rehabilitation and return to sport.	[6, 10, 12-17]	"I remember a few weeks before my first competition, my athletic coach had spent some time giving me information on the possible physical conditions of an athlete who comes from a long time injury and comes back to compete; it was very useful for me to create ideas and expectations about what I would have found in the court."[16]	"It was difficult for me to understand that it was so big, if you know what I mean. And that it would take such a long time."[6]	Barriers and facilitators for psychological readiness to return to sport
**Motivation**				

Descriptive theme (review finding)	Studies contributing to the review finding	Exemplar quotation 1	Exemplar quotation 2	Analytic theme
Receiving feedback, clear communication with and support from trusted people, setting and achieving goals, self-belief and a love of sport/playing sport boosted athletes' motivation. Athletes were typically motivated to return to sport, especially early in the recovery process. Although, losing patience during the long and monotonous rehabilitation depleted their motivation.	[5, 6, 8, 9, 11, 13-20]	"I have had a lot of bad days, a lot of good days. Probably, without my therapist, I wouldn't have made it through."[17]	"If you don't have a relationship with them [physical therapist or athletic trainer], I don't think you'd actually put in the work, and they wouldn't put in the work with you."[13]	Barriers and facilitators for psychological readiness to return to sport
**Confidence**				
Athletes lacked confidence at different stages during recovery from injury, and lacking confidence was a key barrier to returning to sport. When they lacked confidence, athletes responded by self-limiting participation in sport. Regaining confidence was the most important focus of recovery and returning to sport. Having strong support from their social network (including from rehabilitation clinicians) and seeing progress towards their goals during rehabilitation helped athletes build their confidence to return to sport.	[7, 9, 13, 14, 16, 17, 19]	"I feel a little bit more confident because my [physical therapist] told me I would be able to get back to my sport. So, knowing without a doubt I would be able to go back made it all better."[17]	"They just threw me out onto the track and said: 'Go, you have to start some time.' I was not able to get my head round it. I had no confidence."[9]	Barriers and facilitators for psychological readiness to return to sport
**Self-efficacy for recovery from injury**				
During recovery from injury and rehabilitation many athletes lacked self-efficacy and self-esteem. If athletes had previous experience of injury or were an experienced and established member of their team, they were able to draw on these experiences to build resilience during recovery. Previous experience helped athletes know what they needed to do to recover from injury.	[6, 9, 11]	"It's really sad that it happened, but now I have new, bright perspectives and have received answers to many of my questions. This process has been important to me and now I am positive that I will play again."[11]	"But much of it is up to you, or much, everything is up to you. You're the one who decides how well you are going to become."[6]	Barriers and facilitators for psychological readiness to return to sport

Descriptive theme (review finding)	Studies contributing to the review finding	Exemplar quotation 1	Exemplar quotation 2	Analytic theme
**Identity as an athlete and as a member of society**				
Athletes saw themselves as athletes when they were injured, not as patients. Athletes wanted to play sport and felt that playing sport was a central part of who they were as a person. Many athletes had strong self-belief for returning to sport, but were also nervous about their capacity to perform well after returning. For some athletes, their experience of injury had irrevocably changed how they thought of themselves and their place in and contribution to society. Identifying as an athlete was a strong motivating factor for return to sport.	[5, 7-9, 11-14, 19]	"…But I've noticed that she's changed…And of course it's because she's frustrated about what has happened to her. Both from having been told that she was going to be a star and a world talent and that now she was going to conquer the world, and then to all of a sudden sitting at home and not even being able to go for a run. That's a very big and a very dramatic change."[5]	"Like volleyball has always been part of who I am, and so why would you not get back to that or find a way to keep that going for as long as you can?"[13]	Barriers and facilitators for psychological readiness to return to sport
**Priorities for playing sport**				
After experiencing a knee injury, athletes spent time thinking about their preferences and priorities about participating in sport. Athletes recognised that being regularly physically active was beneficial for health. Athletes were aware that there were risks associated with returning to sport after ACL injury. Some athletes decided that the risk for sustaining a new knee injury was not worth the benefit of returning to play their previous sport; other athletes accepted the risk and the need to take active steps to manage risk after returning to their sport.	[8, 13, 14, 18, 20]	"The social aspect, the physical exercise the stress relief and the enjoyment of competitive sport all outweigh that fear and knowledge of the pain and frustration that can come about through re-injury."[19]	"Arthritis is always in the back of my mind…it sucks and that there's just a higher chance of getting it now than ever before…if I want to consider doing football again, it'd [arthritis] probably be in the back of my head."[20]	Barriers and facilitators for psychological readiness to return to sport

References

1. Papaioannou D, Sutton A, Carroll C, Booth A, Wong R. Literature searching for social science systematic reviews: consideration of a range of search techniques. *Health Info Libr J*. 2010;**27**:114-22. PMID: 20565552.

2. Stansfield C, Brunton G, Rees R. Search wide, dig deep: literature searching for qualitative research. An analysis of the publication formats and information sources used for four systematic reviews in public health. *Res Synth Methods*. 2014;**5**:142-51. PMID: 26052653.

3. Sandelowski M, Barroso J. Handbook for Synthesizing Qualitative Research. New York, NY: Springer; 2006.

4. Thomas J, Harden A. Methods for the thematic synthesis of qualitative research in systematic reviews. *BMC Med Res Methodol*. 2008;**8**:45. PMID: 18616818.

5. Thing LF. "Voices of the broken body." The resumption of non-professional female players' sports careers after anterior cruciate ligament injury. The female player's dilemma: is she willing to run the risk? *Scand J Med Sci Sports*. 2006;**16**:364-75. PMID: 16978257.

6. Heijne A, Axelsson K, Werner S, Biguet G. Rehabilitation and recovery after anterior cruciate ligament reconstruction: patients' experiences. *Scand J Med Sci Sports*. 2008;**18**:325-35. PMID: 18067526.

7. Carson F, Polman R. Experiences of professional rugby union players returning to competition following anterior cruciate ligament reconstruction. *Phys Ther Sport*. 2012;**13**:35-40. PMID: 22261429.

8. Tjong VK, Murnaghan ML, Nyhof-Young JM, Ogilvie-Harris DJ. A qualitative investigation of the decision to return to sport after anterior cruciate ligament reconstruction: to play or not to play. *Am J Sports Med*. 2014;**42**:336-42. PMID: 24197615.

9. Nordahl B, Sjöström R, Westin M, Werner S, Alricsson M. Experiences of returning to elite alpine skiing after ACL injury and ACL reconstruction. *Int J Adolesc Med Health*. 2014;**26**:69-77. PMID: 23358350.

10. Gill N, Nagi S. Returning to sport post anterior cruciate ligament reconstruction: a qualitative exploration. *Man Ther*. 2016;**25**:e148.

11. Johnson U, Ivarsson A, Karlsson J, Hägglund M, Waldén M, Börjesson M. Rehabilitation after first-time anterior cruciate ligament injury and reconstruction in female football players: a study of resilience factors. *BMC Sports Sci Med Rehabil*. 2016;**8**:20. PMID: 27429759.

12. Carson F, Polman RCJ. Self-determined motivation in rehabilitating professional rugby union players. *BMC Sports Sci Med Rehabil*. 2017;**9**:2. PMID: 28116102.

13. Burland JP, Toonstra J, Werner JL, Mattacola CG, Howell DM, Howard JS. Decision to return to sport after anterior cruciate ligament reconstruction, part I: a qualitative investigation of psychological factors. *J Athl Train*. 2018;**53**:452-63. PMID: 29505304.

14. Scott SM, Perry MA, Sole G. "Not always a straight path": patients' perspectives following anterior cruciate ligament rupture and reconstruction. *Disabil Rehabil*. 2018;**40**:2311-7. PMID: 28597696.

15. DiSanti J, Lisee C, Erickson K, Bell D, Shingles M, Kuenze C. Perceptions of rehabilitation and return to sport among high school athletes with anterior cruciate ligament reconstruction: a qualitative research study. *J Orthop Sports Phys Ther*. 2018;**48**:951-9. PMID: 29932875.

16. Conti C, di Fronso S, Pivetti M, Robazza C, Podlog L, Bertollo M. Well-come back! Professional basketball players perceptions of psychosocial and behavioral factors influencing a return to pre-injury levels. *Front Psychol*. 2019;**10**:222. PMID: 30800089.

17. Paterno MV, Schmitt LC, Thomas S, Duke N, Russo R, Quatman-Yates CC. Patient and parent perceptions of rehabilitation factors that influence outcomes after anterior cruciate ligament reconstruction and clearance to return to sport in adolescents and young adults. *J Orthop Sports Phys Ther*. 2019;**49**:576-83. PMID: 30759359.

18. Sole G, Mahood C, Gallagher P, Perry M. Overcoming fear of re-injury after anterior cruciate ligament reconstruction: a qualitative study. *J Sci Med Sport*. 2019;**22**:S109.

19. Kunnen M, Dionigi RA, Lichfield C, Moreland A. 'My desire to play was stronger than my fear of re-injury': athlete perspectives of psychological readiness to return to soccer following anterior cruciate ligament reconstruction surgery. *Annals of Leisure Research*. 2020;**23**:447-61.

20. Truong LK, Mosewich AD, Miciak M, Pajkic A, Li LC, Whittaker JL. Balancing return to sport and a functional knee. Exploring the perceptions of exercise therapy and physical activity of youth 1-2 years after a sport-related anterior cruciate ligament injury. *Osteoarthritis Cartilage*. 2020;**28**:S160.
